# Carbapenem-resistant *Klebsiella oxytoca* transmission linked to preoperative shaving in emergency neurosurgery, tracked by rapid detection via chromogenic medium and whole genome sequencing

**DOI:** 10.3389/fcimb.2024.1464411

**Published:** 2024-10-17

**Authors:** Yun-Lan Jiang, Yi-Yu Lyu, Li-Li Liu, Zhi-Ping Li, Dan Liu, Jie-Hao Tai, Xiao-Qian Hu, Wen-Hui Zhang, Wen-Wen Chu, Xue Zhao, Wei Huang, Yi-Le Wu

**Affiliations:** ^1^ Department of Hospital Infection Prevention and Control, Anqing First People’s Hospital of Anhui Medical University, Anqing, Anhui, China; ^2^ Department of Clinical Laboratory, The Second Affiliated Hospital of Anhui Medical University, Hefei, Anhui, China; ^3^ Department of Hospital Infection Prevention and Control, The Second Affiliated Hospital of Anhui Medical University, Hefei, Anhui, China; ^4^ The Fourth Affiliated Hospital of Anhui Medical University, Hefei, Anhui, China; ^5^ Department of Laboratory Medicine, Shenzhen People’s Hospital, The Second Clinical Medical College, Jinan University, Shenzhen, Guangdong, China; ^6^ The First Affiliated Hospital, Southern University of Science and Technology, Shenzhen, Guangdong, China

**Keywords:** surgery, infections, *Klebsiella oxytoca*, carbapenems, whole genome sequencing

## Abstract

**Objectives:**

This study describes the detection and tracking of emergency neurosurgical cross-transmission infections with carbapenem-resistant *Klebsiella oxytoca* (CRKO).

**Methods:**

We conducted an epidemiological investigation and a rapid screening of 66 surveillance samples using the chromogenic selective medium. Two CRKO isolates from infected patients and three from the preoperative shaving razors had similar resistance profiles identified by the clinical laboratory.

**Results:**

The whole genome sequencing (WGS) results identified all isolates as *Klebsiella michiganensis* (a species in the *K. oxytoca* complex) with sequence type 29 (ST29) and carrying resistance genes *bla*_KPC-2_ and *bla*_OXY-5_, as well as IncF plasmids. The pairwise average nucleotide identity values of 5 isolates ranged from 99.993% to 99.999%. Moreover, these isolates displayed a maximum genetic difference of 3 among 5,229 targets in the core genome multilocus sequence typing scheme, and the razors were confirmed as the contamination source. After the implementation of controls and standardized shaving procedures, no new CRKO infections occurred.

**Conclusion:**

Contaminated razors can be sources of neurosurgical site infections with CRKO, and standard shaving procedures need to be established. Chromogenic selective medium can help rapidly identify targeted pathogens, and WGS technologies are effective mean in tracking the transmission source in an epidemic or outbreak investigation. Our findings increase the understanding of microbial transmission in surgery to improve patient care quality.

## 1 Introduction

Surgical site infections (SSIs), the most prevalent healthcare-associated infections, occur in approximately 0.5% to 3% of surgery patients (Seidelman et al., 2023). Post-neurosurgical infections are a common and very harmful complication requiring re-operation, prolonging hospital stay lengths, and increasing disability and mortality rates (Li et al., 2023). Infections with antibiotic-resistant bacteria after neurosurgery have become an important treatment challenge due to the low blood-brain barrier permeation rate of most antibiotics (Li et al., 2023). Carbapenems are a potent class of broad-spectrum antibiotics with strong antibacterial activity and are considered “last-line” antibiotics (Tompkins and van Duin, 2021). Carbapenem-resistant organisms have emerged and spread worldwide (Nordmann et al., 2011). The World Health Organization (WHO) has classified these organisms as an urgent public health threat (Wyres and Holt, 2022). Carbapenem-resistant organisms are usually multidrug-resistant, extensively drug resistant, and even pandrug-resistant bacteria (Li et al., 2023). The growing prevalence and the considerable morbidity and mortality of carbapenem-resistant organisms have attracted increasing attention in neurosurgery (Li et al., 2023).

*Klebsiella* species often cause respiratory, urinary tract, and wound infections, and they are increasingly recognized as pathogens of emerging nosocomial infections and outbreaks, following *Klebsiella pneumoniae* (Guzmán-Puche et al., 2021; Neog et al., 2021). *K. oxytoca* has been associated with neurosurgical procedures (Tang and Chen, 1995). Recently, with the emergence of bacterial resistance to carbapenems, carbapenem-resistant *K. oxytoca* (CRKO) has been identified as a species associated with neurosurgical procedures (Guanghui et al., 2020); however, transmission routes of CRKO in neurosurgery have rarely been reported.

Whole genome sequencing (WGS) technologies provide rich data and capture numerous features of isolates; data helpful for identifying resistance and virulence genes, mobilizable plasmids, and evolutionary information, as well as for epidemiological tracking and pathogen surveillance (Mustafa, 2024). This study investigated neurosurgical cross-transmission infections with CRKO by applying rapid chromogenic selective medium and WGS technologies to track the source of cross-transmission and improve surgical patient care.

## 2 Materials and methods

### 2.1 Setting and epidemiological investigation

This investigation was conducted in a tertiary teaching hospital with 1,460 beds in Anqing (Anhui Province of China). Two patients with SSIs were reported in the neurosurgical intensive care unit (ICU) on October 8, 2023 after emergency neurosurgery. Two CRKO isolates with similar resistance profiles were isolated from the neurosurgical sites of these two infected patients, and an cross-transmission was suspected. We investigated the transmission and conducted an active surveillance to detect the source of infections and control the spread. The Ethical Committee of The Second Affiliated Hospital of Anhui Medical University approved the study protocol (YX2023-102).

### 2.2 Active surveillance cultures and microbiological methods

According to information from epidemiological investigation and interviews of medical staff in the operating room and neurosurgical ICU, we collected active surveillance samples from the potential related patients, medical staff (healthcare workers, shaver, cleaners), neurosurgery disinfectants, shared environmental and device surfaces (hanging towers, bedrails, infusion pumps, cardiovascular monitors, etc.), and equipment for preoperative shaving (such as razors and shampoo) in the operating room and neurosurgical ICU involved. We inoculated CHROMagar mSuperCARBA (CHROMagar™, France) medium, a chromogenic selective medium with 95.6% to 96.5% sensitivity to detect carbapenemase producers, with surveillance samples to rapidly screen for carbapenemase-producing organisms (Alizadeh et al., 2018). After 24 h of culture at 35°C ± 2°C, the blue colonies growing on the selective agar suggested the potential presence of CRKO. Then the suspected isolates were further identified via matrix-assisted laser desorption/ionization time-of-flight mass spectrometry (MALDI-TOF MS, Germany). Antimicrobial susceptibility was tested using the VITEK2 drug sensitivity analyzer (bioMérieux, Marcy L’Étoile, France), and *K. pneumoniae* ATCC BAA-1705 and *Escherichia coli* ATCC 25922 as quality control strains. Testing results were interpreted referring to the performance standards of the Clinical and Laboratory Standards Institute (CLSI) guidelines (Wayne, 2019).

### 2.3 Whole genome sequencing and genome-based analysis

WGS was performed for detected CRKO isolates from patients with SSIs and from surveillance samples. The concentration and quality of genomic DNA of these isolates extracted by the cetyltrimethylammonium bromide (CTAB) method were determined using a Qubit fluorometer (Invitrogen, USA) and a NanoDrop spectrophotometer (Thermo Scientific, USA). Sequencing libraries were generated using the TruSeq DNA Sample Preparation Kit (Illumina, USA) and the Template Prep Kit (Pacific Biosciences, USA). WGS was ran on an Illumina Novaseq platform at Personal Biotechnology Company (Shanghai, China). After using AdapterRemoval and SOAPec to remove adapter sequences and filter data (Lindgreen, 2012; Luo et al., 2012), the reads were assembled by the SPAdes and A5-miseq for constructing scaffolds and contigs (Bankevich et al., 2012; Coil et al., 2015). Finally, the genome sequences were obtained after correction using the Pilon software (Walker et al., 2014). Species was identified according to the PubMLST website (https://pubmlst.org/). Multilocus sequence typing (MLST) was performed via the MLST 2.0 at the Center for Genomic Epidemiology (https://cge.food.dtu.dk/services/MLST/). Resistance genes were determined using ResFinder (http://genepi.food.dtu.dk/resfinder). Plasmids were identified with PlasmidFinder (https://cge.food.dtu.dk/services/PlasmidFinder/). Virulence factors were determined in the virulence factor database (http://www.mgc.ac.cn/cgi-bin/VFs/v5/main.cgi). Pair-wise average nucleotide identity (ANI) values were calculated using the orthologous average nucleotide identity tool (OAT), using the proposed cut-off value of 95.0–96.0% for species demarcation (Lee et al., 2016). In addition, SeqSphere+ version 10.0 (Ridom, Münster, Germany) (https://www.ridom.de/news/) and the publicly available core genome MLST (cgMLST) scheme for *K. oxytoca* were used to calculate the minimum spanning tree (MST). A *Klebsiella michiganensis* isolate “Control”, which was isolated from a patient in the ICU of the same hospital on October 21, 2023, was used as the reference strain in the cgMLST analysis. Isolates with less than 9 allelic differences from 5,229 targets of the cgMLST scheme were defined as highly related (Dabernig-Heinz et al., 2024).

## 3 Results

### 3.1 Transmission investigation

Surveillance data indicated that none of the 36 patients who underwent neurosurgery in August 2023 developed SSIs. However, two of 23 patients (8.7%) undergoing neurosurgery in the neurosurgical ICU developed SSIs due to CRKO in September 2023. The two CRKO isolates with similar resistance profiles were isolated from cerebrospinal fluid and wound effusion in patients who had undergone emergency neurosurgery on September 27, 2023. Detailed characteristics of the two patients are presented in **Table 1**.

**Table 1:** Epidemiological and clinical characteristics of 5 Carbapenem-resistant *Klebsiella oxytoca* (*CRKO*) isolates from patients and razors.

No. of isolates	Sample source	Gender	Age	Clinical diagnosis	Admission /operation date	Invasive procedure	Sample date	Sequence type
P1	Patient1 (cerebrospinal fluid)	Male	75 y	Closed encephalon injury	27/09/2023	Ventriculostomy	04/10/2023	ST 29
P2	Patient2 (wound effusion)	Male	69 y	Ruptured cerebral aneurysm	27/09/2023	Intracranial aneurysm surgery	04/10/2023	ST 29
R1	An electric razor	−	−	−	−	−	08/10/2023	ST 29
R2	A charging cable of the electric razor	−	−	−	−	−	08/10/2023	ST 29
R3	An unused razor head ^a^	−	−	−	−	−	08/10/2023	ST 29

a An unused razor head with incomplete packaging.

We collected 66 samples through active surveillance and detected 10 isolates (**Table 2**). Among them, three CRKO isolates were obtained from a preoperative electric shaving razor, its charging cable, and an unused razor head with an incomplete packaging. The traditional preoperative shaving procedure in the hospital involved using a unique electric razor head for each patient, and disinfecting the handle of the electric razor before each use. Besides, any unused razor heads and disinfected handles were stored together. We discovered that the two patients with SSIs both had undergone preoperative shaving with these electric razors. Therefore, we suspected contaminated razors during preoperative shaving procedure as the source of transmission.

**Table 2:** Results of environment, healthcare worker, and patient sampling during this transmission investigation.

Sampling site	Sampling number	Culture positive (%)	Bacterial species
Healthcare workers (medical personnel, shavers, cleaners)			
Throat and rectal swabs	18	0 (0.0)	None
Hands	8	0 (0.0)	None
Telephone and watch of shaver	2	2 (100.0)	1 *Acinetobacter baumannii*, 1 *Klebsiella pneumoniae*
Patients and possible epidemiological link			
Rectal swabs	7	2 (28.6)	1 *Klebsiella pneumoniae*, 1 *Escherichia coli*
Dress for family visits	2	0 (0.0)	None
Environment and equipment			
Hand hygiene facilities in the nurse station	2	0 (0.0)	None
Disinfectant in the operating room	1	0 (0.0)	None
Instruments of bed surroundings (Infusion pumps, cardiovascular monitors, etc.)	7	2 (28.6)	1 *Acinetobacter baumannii*, 1 *Klebsiella pneumoniae*
Noninstruments of bed surroundings (hanging tower, bedrails)	2	0 (0.0)	None
Tools for shaving			
Electric razors	9	4 (44.4)	3 carbapenem*-resistant Klebsiella oxytoca*, 1 *Acinetobacter baumannii*
A container for razors, dress for shaver, and packaging of shampoo	8	0 (0.0)	None
*Total*	n=66	10 (15.2)	

### 3.2 Implementation of control measures

Upon confirmation of CRKO infections, the two patients were immediately isolated and treated with antibiotic therapy. While investigating the transmission, additional infection control procedures were strengthened including hand hygiene, cleaning, disinfection, sterilization, surveillance cultures, and re-education of healthcare staff. Following the detection of CRKO isolates solely on the razors, when necessary, trained nurses were instructed to use disposable clippers for preoperative shaving. These cut hair close to the skin, leaving a short stubble without touching the skin. Moreover, the department of hospital infection prevention and control regularly supervised standardized preparation procedures. Finally, after the implementation of these control measures, no other patients developed SSIs with CRKO. The two infected patients were successfully treated and discharged.

### 3.3 Analysis of antibiotic susceptibility, resistance and virulence genes, plasmids, and genetic relatedness

**Figure 1:**
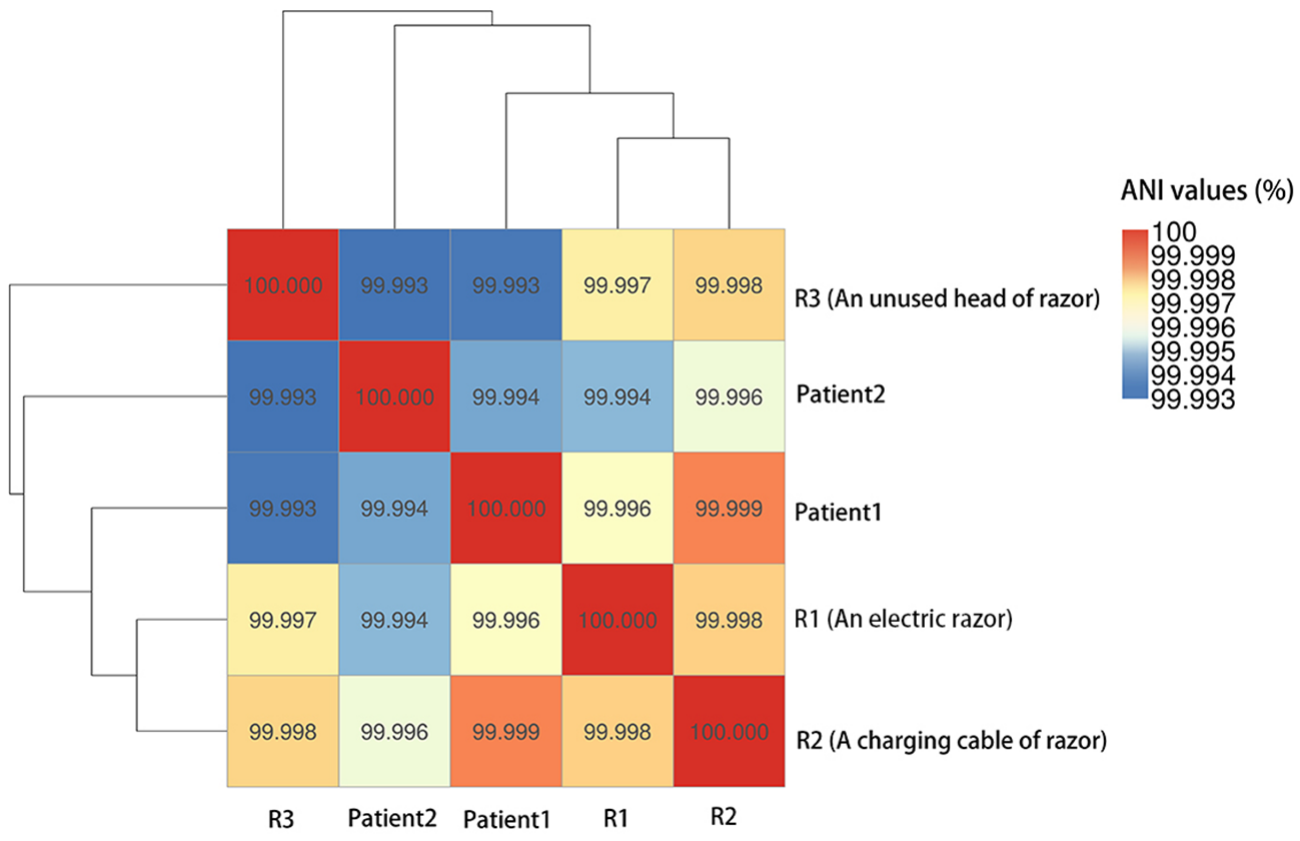
Pairwise average nucleotide identities (ANI) values among the genomes of the 5 CRKO isolates from infected patients and razors.

**Figure 2:**
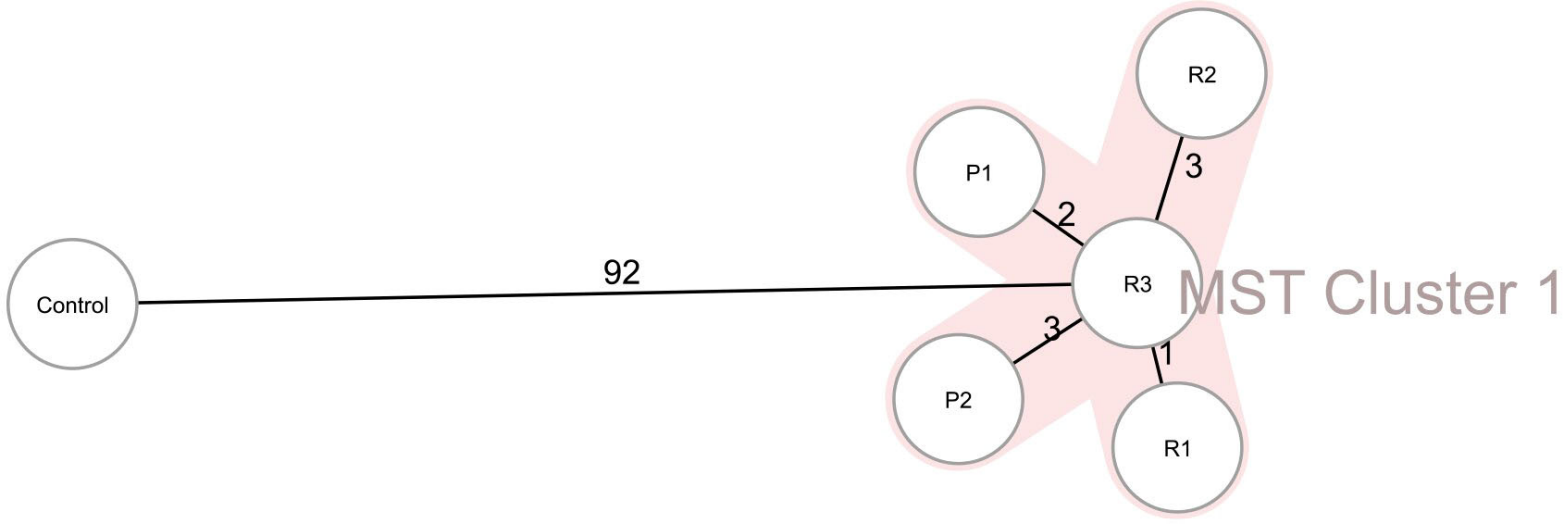
Ridom SeqSphere+ minimum spanning tree (MST) for 5 CRKO isolates from infected patients and razors based on 5,229 target genes (2536 cgMLST and 2693 accessory) with missing values are an own category. MST Cluster distance threshold: 9. P1, Patient 1; P2, Patient 2; R1, Razor 1; R2, Razor 2; R3, Razor 3.

Two CRKO isolates from infected patients and three from razors had consistent resistance patterns based on the antibiotic susceptibility testing results identified by the clinical laboratory. All the isolates were resistant to ticarcillin/clavulanic acid, piperacillin/tazobactam, aztreonam, imipenem, and meropenem (**Table 3**). WGS results identified these isolates as *K. michiganensis* (a species in the *K. oxytoca* complex) with sequence type 29 (ST29). All isolates carried two resistance genes (*bla*_KPC-2_ and *bla*_OXY-5_), as well as IncF plasmids (IncFIB(K) and IncFII(K)). Two isolates carried 67 virulence genes, and three isolates carried 66 virulence genes but lacked *stbA* (**Table 4**). The pairwise ANI values among the genomes of the 5 isolates ranged from 99.993% to 99.999% (**Figure 1**). Besides, the minimum spanning tree displayed a maximum difference of 3 among 5,229 targets in the core genome scheme between 5 isolates, whereas these 5 isolates had a significant allele difference (more than 92) to the reference strain “Control” (**Figure 2**), revealing the close relatedness between the isolates from patients and razors.

**Table 3:** Antimicrobial resistance phenotypes, resistance genes, and plasmids of 5 CRKO isolates from infected patients and razors.

Sample source	Antimicrobial susceptibility																	Resistance gene		Plasmid	
	TIM	TZP	ATM	IMP	MEM	AMK	CAZ	CFP/SU	FEP	TOB	CIP	LEV	DO	MH	TGC	CT	SXT	KPC-2	OXY-5	IncFIB(K)	IncFII(K)
P1	R	R	R	R	R	S	S	S	S	S	I	I	S	S	S	S	S	+	+	+	+
P2	R	R	R	R	R	S	S	S	S	S	I	I	S	S	S	S	S	+	+	+	+
R1	R	R	R	R	R	S	S	S	S	S	I	I	S	S	S	S	S	+	+	+	+
R2	R	R	R	R	R	S	S	S	S	S	I	I	S	S	S	S	S	+	+	+	+
R3	R	R	R	R	R	S	S	S	S	S	I	I	S	S	S	S	S	+	+	+	+

P1, Patient 1; P2, Patient 2; R1, Razor1; R2, Razor 2; R3, Razor 3.

TIM, ticarcillin/clavulanic acid; TZP, piperacillin/tazobactam; ATM, aztreonam; IMP, imipenem; MEM, meropenem; AMK, amikacin; CAZ, ceftazidime; CFP/SU, cefoperazone/sulbactam; FEP, cefepime; TOB, tobramycin; CIP, ciprofloxacin; LEV, levofloxacin; DO, doxycycline; MH, minocycline; TGC, tigecycline; CT, colistin; SXT, trimethoprim/sulfamethoxazole; R, resistant; S, susceptible; I, intermediate.

**Table 4:** Virulence factors of 5 CRKO isolates from infected patients and razors.

Virulence factors class	Related genes	P1	P2	R1	R2	R3
Adherence	*mrkABCDFHIJ*	+	+	+	+	+
	*fimABCDEFGHIK*	+	+	+	+	+
Antiphagocytosis	*Capsule*	+	+	+	+	+
Efflux pump	*acrAB*	+	+	+	+	+
Iron uptake	*iutA*	+	+	+	+	+
	*entABCDEFS*	+	+	+	+	+
	*fepABCDG*	+	+	+	+	+
	*fes*	+	+	+	+	+
	*iroE*	+	+	+	+	+
Regulation	*rcsAB*	+	+	+	+	+
Secretion system	*clpV/tssH*	+	+	+	+	+
	*hcp/tssD*	+	+	+	+	+
	*impA/tssA*	+	+	+	+	+
	*sciN/tssJ*	+	+	+	+	+
	*vipA/tssB*	+	+	+	+	+
	*vipB/tssC*	+	+	+	+	+
	*clpV*	+	+	+	+	+
	*dotU*	+	+	+	+	+
	*icmF*	+	+	+	+	+
	*impAFGHJ*	+	+	+	+	+
	*ompA*	+	+	+	+	+
	*sciN*	+	+	+	+	+
	*vgrG*	+	+	+	+	+
	*SCI-I T6SS*	+	+	+	+	+
Serum resistance	*LPS rfb locus*	+	+	+	+	+
Biofilm formation	*adeG*	+	+	+	+	+
Cell surface components	*sugC*	+	+	+	+	+
Endotoxin	*lpxC*	+	+	+	+	+
Enzyme	*eno*	+	+	+	+	+
Fimbrial adherence determinants	*stbA*	−	−	+	−	+
	*stbCD*	+	+	+	+	+
	*stiB*	+	+	+	+	+
Magnesium uptake	*mgtB*	+	+	+	+	+
Protease	*pla*	+	+	+	+	+

P1, Patient 1; P2, Patient 2; R1, Razor 1; R2, Razor 2; R3, Razor 3.

## 4 Discussion

Despite great advances in neurosurgical technology, SSIs after neurosurgery remain a common problem (Velnar et al., 2023). The incidence of SSIs post-craniotomy ranges between 5.1% and 6.2% (He et al., 2024; Lee et al., 2024). Post-craniotomy SSIs can significantly impact patients recovery and lead to increased morbidity, mortality, hospital stay lengths, pain, and costs, and require re-operation (Velnar et al., 2023). Cerebrospinal fluid leaks, infratentorial surgery, emergency surgery, re-operation, and other factors have been associated with post-craniotomy SSIs (Magni et al., 2023). SSIs can be cause by bacteria from the patients themselves or from medical staff, equipment, or the environment entering surgical incision sites (Tanner and Melen, 2021). One previous study has reported a carbapenemase-producing *K. pneumonia* outbreak caused by a barber’s contaminated shaving razor (Dai et al., 2014). Despite *K. oxytoca* being the 2^nd^ most clinically prevalent *Klebsiella* species, there is limited information about it, especially about the carbapenem-resistant species and its infectivity and epidemiology in neurosurgery (Neog et al., 2021). According to data from the worldwide bacterial collection of SENTRY program, the resistance rate of *K. oxytoca* to carbapenems is 1.8% with an alarming increasing rate (Yang et al., 2022). *K. oxytoca* is known to cause healthcare associated infections outbreaks (Yang et al., 2022). In this study, the CRKO isolates from infected patients and the razors shared the same resistance profiles, and had pairwise ANI values higher than 99.993% and a maximum difference of 3 among 5229 targets in the core genome scheme, indicating strong relatedness. A cgMLST analysis may enhance the MLST analysis by assigning allele numbers correlated to the exact genetic sequence of thousands of coding regions throughout the genome, providing a very high resolution and facilitating transmission and outbreak investigations (Dabernig-Heinz et al., 2024). Our findings confirm that contaminated razors can be the sources of CRKO transmission. Thus, inadequate preoperative shaving procedures can cause neurosurgical site infections. Moreover, *K. oxytoca* seems well adapted to healthcare environments (Yang et al., 2022), and implementation of sanitation protocols, equipment handling, aseptic technique, and antibiotic stewardship strategies should be emphasized, especially in preparation of surgical procedures.

Although the benefits of hair removal for preventing SSIs remain controversial in the literature, preoperative shaving at the intended surgical incision site is a traditional practice that reduces the interference of hair at the surgical site and incision suture, promoting cleanliness and facilitating dressing applications (Kose et al., 2016; Tanner and Melen, 2021; Liu et al., 2022). Preoperative hair removal may result in skin trauma, which in turn contributes to SSIs (Tanner and Melen, 2021). A meta-analysis has indicated that, when compared to the SSIs risk in patients without hair removal, the risk increases in patients who undergo hair removal using a razor (RR: 1.82) (Tanner and Melen, 2021). WHO Guidelines recommend that hair should not be routinely removed in patients undergoing surgical procedures, and that it should be removed with a clipper if absolutely necessary (World Health Organization).

Numerous advanced techniques have been applied for the prevention and control of infections and for promoting patient care. Timely evaluation of transmission sources is critical for implementing intervention strategies to contain the spread. In this study, we successfully applied rapid chromogenic screening and WGS technologies to investigate and control the transmission, and these practicabilities were well confirmed. We used chromogenic-based selective medium to screen for carbapenemase-producing organisms from active surveillance samples, and rapidly detected three suspected CRKO isolates on the razors (within 24 hours) to help timely implementation of precise control measures. The range of available chromogenic culture medium has experienced a rapid expansion. Chromogenic medium are based on synthetic chromogenic enzyme substrates to target specific species (Perry, 2017). Compared with traditional culture medium, chromogenic medium often save costs by reducing labor time and reagents use, while contributing to rapid pathogens identifications (Perry, 2017).

WGS technologies have contributed to the diagnosis, treatment, surveillance, and epidemiological investigations of bacterial infections (Mustafa, 2024). Utilizing WGS and genome-based analysis, these *K. oxytoca* isolates were identified as the species of *K. michiganensis* with ST29. *K. oxytoca* is described as a complex of nine species including *K. michiganensis* (Yang et al., 2022). *K. michiganensis* was first recovered from a toothbrush holder in 2012 (Saha et al., 2013). However, the clinical significance including the prevalence, disease spectrum, and severity of each species in the *K. oxytoca* complex remains largely unknown (Yang et al., 2022). By applying a genome-based analysis, we achieved the precise species identification, providing comprehensive information on the species in the *K. oxytoca* complex as well as on patient treatment, surveillance, and infection control interventions (Yang et al., 2022). We found that the isolates carried *bla*_KPC-2_ and *bla*_OXY-5_ resistance genes and IncF plasmids. In addition to the intrinsic *bla*_OXY_ of *K. oxytoca*, a number of genes encoding *β*-lactamases have been reported, and *bla*_KPC-2_ is the most common carbapenemase gene and is often carried on IncF plasmids (Yang et al., 2022). The horizontal transmission of *bla*_KPC-2_-carrying plasmids across different genera of bacteria has been demonstrated (Schweizer et al., 2019). We also identified virulence factors such as *mrk*, *fim*, *iutA*, and *iroE* genes in these isolates. The *mrk* gene cluster can encode mannose-resistant *Klebsiella*-like hemagglutinins, allowing *Klebsiella* spp. to attach to surfaces and form biofilms (Yang et al., 2022). Particularly, the *iut*A gene seems to be important for hypervirulence (Lee et al., 2017). Based on our findings, we believe that *K. oxytoca* can become a potential major threat to public health due to their ability to acquire resistance to antimicrobials and carry a large number of virulence genes (Yang et al., 2022). Supplemented with epidemiological data, we further validated cgMLST as a useful tool for investigating the source and route of pathogen transmission. cgMLST had been applied to guide interventions, determine durations, and evaluate effects of interventions strategies for other pathogens (Kjær Hansen et al., 2021; Knudsen et al., 2022). Also based on our results, WGS technologies are effective to precisely track transmission sources and provide abundant information on the complete genome of pathogens in an epidemic or outbreak investigation.

Increase in the numbers of multidrug-resistant bacteria, particularly of carbapenem-resistant organisms, have proved fatal to patients undergoing neurosurgery (Li et al., 2023). The surgical team and any professionals directly providing surgical care have an important responsibility to prevent SSIs (World Health Organization; Tvedt et al., 2017). The safety of surgical patients is of the utmost importance, and a list of evidence-based best practices including hand hygiene, preoperative skin antisepsis, antimicrobial irrigation, and others are routinely employed to promote a safe outcomes for surgical patients (Bashaw and Keister, 2019). Evidence-based information is needed to clarify the risk factors of SSIs and improve the quality of care (World Health Organization, 2018). With this study based on WGS, we demonstrated that contaminated razors can lead to SSIs with carbapenem-resistant organisms; therefore, a standard preoperative shaving procedure should be established and executed. Moreover, updated techniques such as chromogenic selective medium and WGS technology can be applied for prevention and control of SSIs and promoting surgical patient care.

We are aware of our study’s limitations. First, although several virulence genes such as the hypervirulence *iut*A were detected via WGS, the virulence phenotypes were not tested. Second, although we confirmed that razors can transmit pathogens, including carbapenem-resistant organisms, and lead to SSIs, the survival time of these pathogens in the environment such as in razors needs to be further investigated. Finally, the cross-transmission involved a small number of isolates with the same sequence type. Therefore, further research is needed to account for the genetic diversity of CRKO complex bacteria.

To conclude, a cross-transmission of CRKO infections caused by contaminated preoperative shaving razors was confirmed during emergency neurosurgery, and revealed that contaminated shaving razors can transmit CRKO. Whenever hair removal is necessary, it should be removed in the proper area, and clippers may be a more proper alternative to razors (Knudsen et al., 2022; Seidelman et al., 2023). Chromogenic-based selective medium is a sensitive and convenient means to rapidly detect suspected isolates of carbapenem-resistant organisms in an epidemic or outbreak investigation (Alizadeh et al., 2018). We recommend applying WGS technology to track the microbial movement from the original to the infection site. Findings of this study will contribute to the understanding of microbial transmission in surgery and improving of infection prevention and control and patient care.

## Data Availability

The raw sequence data in this study can be found in the online repository. The name of the repository and accession number can be found below: NCBI Sequence Read Archive, PRJNA1171638.
